# Caregiver experience of specialist hospice palliative care in rural communities: A qualitative study exploring rural culture, hospice nurse characteristics, roles, and carer strategic ideas

**DOI:** 10.1177/26323524251332970

**Published:** 2025-04-22

**Authors:** Rowan Daniel Beaumont Bell, Caren Amanda Barnett, Matthew Joseph Wilson, Philippa Marie Twigg

**Affiliations:** 1 Hospice Waikato, Hamilton, New Zealand

**Keywords:** qualitative research, caregivers, palliative care, rural populations, specialists, hospices, culture, nurses, role

## Abstract

**Background:**

Barriers, facilitators, and inequity of access to rural palliative care have been explored from caregiver and healthcare worker perspectives. However, caregiver insights into specialist (secondary) hospice palliative care are sparse. This study facilitates rural people who cared for patients at end-of-life a chance to reflect on experiences and suggest improvements to specialist support in rural communities.

**Objectives:**

To explore how caregivers perceive a specialist hospice palliative care service, and how it could adapt to rural community needs.

**Design:**

Semi-structured interviews over 6 months with bereaved carers of patients under specialist hospice care. A general inductive approach to data analysis.

**Methods:**

Hospice Waikato; a regional specialist palliative care service. Carers were ineligible if bereaved <6 months and must have had service input within 2 years. Those with complex grief were excluded. Twenty-four participants were interviewed.

**Results:**

Themes were: 1. *Rural culture*; end-of-life care challenges and adaptations. 2. *Positive hospice nursing characteristics*; empathy, holism, and emotional support. 3. *Role of the hospice nurse*; was identified. 4. *Strategic ideas*; embedded community practitioners, increased resources for emotional support and weekends, and timely access to equipment.

**Conclusion:**

Adaptation is required by both carers and the specialist hospice team to overcome rural inequity. A healthcare navigator was desired with the suggestion that this could be the specialist hospice nurse. There is a need to explore the balance between tailoring care to different rural communities and providing consistent and equitable care. Future research could study this and/or compare stakeholder perceptions of different rural palliative care models.

## Background

Inequity in access to palliative care in rural communities is well described. Reasons proposed for discrepancy include distance from specialist centres, education (of healthcare professionals and patients/carers), available healthcare resources, barriers in communication between care teams, and rural community propensity to have low expectations.^1–4^ Economies of scale also mean a lack of equipment and infrastructure in rural settings.^5^

Access and issues with the provision of rural palliative care are well documented around the world.^1–3,6–10^ In developing countries, this phenomenon is heightened with most palliative care limited to urban centres and still very much in the early phases of development.^11^ Paucity of essential medicines, such as opioids, is common and this challenge is increased in rural settings.^12^

It is deemed important to provide palliative care within rural communities rather than bringing the patient to urban centres with community-driven approaches providing good outcomes in rural areas.^13,14^ The use of telehealth as well as communicating with and utilising the skills of primary caregivers are also important.^14,15^ Success has also been had with weekly multi-disciplinary team discussions and telehealth may also be a tool to upskill and support caregivers.^14^

Rural healthcare tends to be more generalist and perhaps for this reason, carers report a need for designated palliative care healthcare professionals with clearly defined roles and responsibilities.^16^ Building collaborative relationships with primary healthcare professionals and examining how trained specialists can support them is paramount.^15^ Additionally, having locally practising nurses as case coordinators has been suggested to improve care.^2,3^

Knowledge of the concept of palliative care is lacking among rural patients and carers.^1^ The impact of rurality on the preparedness of caregivers highlighted that they felt under-skilled for a metaphorical “battle” and isolation was described throughout the caregiving journey. However, positive experiences were illustrated with “fostering of closer relationships” and feeling valued.^16^

Clearly, the existing literature examines palliative care in rural communities from several viewpoints, but there is very sparse data from the point of view of bereaved caregivers who have lived through caring for the dying and have had support from specialist (secondary) hospice palliative care services.

## Objectives

This research will explore bereaved rural caregiver perceptions of a specialist hospice palliative care service provided to them and the people that they cared for. It will identify how to improve support to rural communities, giving carers an opportunity to reflect on challenges and positive experiences; the goal being to think strategically about how these points of view could lead to enhanced palliative care support.

## Design

This is a qualitative research study, taking a general inductive approach to data analysis.^17^ Data were collected from 24 participants during 23 semi-structured interviews with bereaved carers of patients under specialist hospice care. Participants were based in rural areas of the Waikato region of New Zealand. Interviews took place over a 6-month period from 27 November 2023 to 23 May 2024.

## Setting

Hospice Waikato is a community hospice in Hamilton, New Zealand. It provides specialist palliative care to approximately 500,000 people across Waikato, a region of approximately 24,000 km^2^.

Over 50% of the patients under the care of Hospice Waikato are based outside Hamilton city in rural locations. These areas are diverse, comprising of small towns, remote coastal settings, farmland, hills, and mountains. Distance from the outermost reaches of the region to the hospice ranges from 200 km to the North, 250 km to the South, 150 km to the West, and 90 km to the East.

In the case of Hospice Waikato, specialist (secondary) palliative care is defined by referral criteria.^18^ Practically, it is characterised by the primary healthcare provider’s need for support. If there is doubt as to specialist palliative care requirement, a full nursing assessment is arranged.

Each rural patient is allocated to the corresponding hospice nurse covering the area. Some of these nurses live in the communities in which they work, whereas others commute. They work alongside primary practitioners including district nurses and general practitioners, providing specialist nursing oversight and liaison.

## Methods

The reporting of this study conforms to the Standards for Reporting Qualitative Research (SRQR) EQUATOR guideline^19^ (Supplemental File 1).

### Recruitment and sampling strategy

Purposive, “expert” sampling was undertaken between 3 November 2023 and 24 April 2024. Hospice Waikato rural nurses were asked to provide names of people they felt would have “something to say” as primary caregivers (“experts”) to a deceased hospice patient. This method of recruitment, widely used in qualitative research,^20^ was used to select information-rich cases to provide more meaningful feedback, with the added practicality that these carers were felt to be more likely to be willing to participate.

Participants must have had input with the service in the last 2 years, with an exclusion time of 6 months post-index patient death to allow normal grieving.

Potential participants were contacted by the research team and sent a Participant Information Leaflet. Once consent was received, they were allocated a participant number.

The Hospice Waikato Kaiāwhina (Māori cultural liaison) made initial contact with potential Māori participants and offered ongoing cultural support.

Health New Zealand, Te Whatu Ora Waikato, and Te Puna Oranga Māori Research Review Committee (MRRC) consultation encouraged the researchers to actively recruit equal numbers of Māori and non-Māori, with a view to promoting equity.

After 12 interviews, only one-third were Māori ethnicity, so the research team requested further Māori participants be put forward. Recruitment ended with 24 interviewees, 61% non-Māori and 39% Māori.

### Data collection

Four volunteers identified by the Hospice Volunteer Services Manager attended a training day with the research team. They all live rurally. Two were retired nurses, one with professional palliative care experience, the other in public health. The others were a retired palliative care psychotherapist and a journalist. Volunteers rather than researchers were chosen to conduct interviews primarily for practicality, convenience, and logistical reasons. Reflexivity and neutrality were also considerations as volunteers would not have some of the bias of working directly within the service or with any of the index patients or caregivers.

The volunteers confirmed participant consent in writing or verbally. Demographic data were also collected. If primary participants felt support and information from other significant family members was important, then it was acceptable to have them contribute to a combined interview. The volunteers conducted 23 semi-structured interviews with primary caregivers. One interview was contributed to by a secondary carer. Interviews were offered either in person or via telephone depending on participant preference. The purpose was to answer the following overarching question and sub-questions designed across a series of meetings by the research team (Table 1).

**Table 1.** Interview questions.

*Thinking back to your experience do you have any suggestions on how the care for dying patients and their whānau (family) in your local area could be improved?* - What is your understanding of the role of Hospice Waikato within your community? - What positive experiences did you and your relative have with hospice/palliative care? - What struggles did you or your relative face during caring for your loved one and how could you have been helped more? - Were there areas of hospice care that were lacking? - Are there any ways that you can see how hospice/palliative care could be changed to fit better with your local area? - As a local person is there anything particularly important that we should know about your area and its people that would help to develop hospice services here?

Sub-questions were designed by the research team in a series of meetings to provide interviewers with prompts.

The overarching question and sub-questions were designed to achieve the following:

1. Promote meaningful feedback from participants on the hospice service based on their experience of being a carer in their local, rural area.
2. To address the sparsity of data in the literature on perceptions of rural caregivers of specialist hospice care and to understand how living rurally might affect care.

## Analysis

The interviews were audio recorded and transcribed verbatim by a team of hospice volunteers.

### Demographics

Demographic data are displayed in Tables 2 and 3. Of note, 74% of participants were female. This may be explained by the carer role being taken on by women more frequently than men in the son/daughter subgroup of which 8/9 (89%) participants were female.

**Table 2.** Primary participant characteristics.

Demographic	Number (%)
Gender	
Male	6 (26)
Female	17 (74)
Ethnicity	
European	14 (61)
Māori	9 (39)
Age	
18–24	0 (0)
25–34	1 (4)
35–44	1 (4)
45–54	3 (13)
55–64	9 (39)
65–74	5 (22)
75+	4 (18)
Relationship to care-receiver	
Spouse/partner	11 (48)
Parent/guardian	1 (4)
Son/daughter	9 (40)
Sibling	1 (4)
Friend	0 (0)
Other	1 (4)
Interview type	
In person	17 (74)
Telephone	6 (26)
Time between patient death and interview	
Mean	14 months
Median	12 months
Range	6–23 months

**Table 3.** Index patient characteristics.

Demographic	Number (%)
Gender	
Male	9 (39)
Female	14 (61)
Ethnicity	
European	12 (52)
Māori	10 (44)
Middle Eastern/Latin American/African	1 (4)
Age at death	
Mean	71 years
Median	74 years
Range	43–98 years
Place of death	
Home	18 (78)
Hospital	2 (9)
Hospice inpatient unit	2 (9)
Age residential care facility	1 (4)
Diagnosis	
Cancer	19 (83)
Non-cancer	4 (17)
Duration of specialist hospice care	
Mean	6 months
Median	5 months
Range	1–17 months
Distance to specialist palliative care centre by road	
Mean	116 km
Median	102 km
Range	28–258 km
Dates range of index patient death	18 December 2021–24 August 2023

### Data analysis

Data analysis followed a general inductive approach.^17^ Two of the research team, RBB and CB independently verified the transcripts alongside the original audio and coded the data using NVivo (2020) (a Lumivero research software). Subsequently, they met to compare codes and formulate hierarchical frames. The researchers re-immersed themselves in the data to identify text that best reflected the codes and frames and refined categories further, creating the key themes. This iterative process facilitated immersion and familiarisation with the data as well as confidence that data saturation was achieved.

Verification of themes occurred in discussion with the other members of the research team. Two volunteer interviewers attended a designated meeting to discuss the themes providing additional corroboration.

### Bereavement care

Carers who felt to have complex grief were excluded. If a participant experienced emotional distress related to taking part in the study, the Hospice Waikato counselling team offered support and referral on to the appropriate community counselling service.

## Results

Four key themes were identified. Rural Culture, Positive Hospice Nurse Characteristics, the Role of the Specialist Hospice Nurse, and Strategic Ideas.

### Rural culture

Remote living, carer challenges, and adaptation were identified as themes describing the interplay between rural culture and end-of-life care.

Remoteness was a challenge in caring for a person at the end of life in terms of access to specialist hospice care and healthcare in general.

P4: “I guess there’s always that sense of. . . isolation and that care is a long way away. . .”

Participants acknowledged that living in an isolated area meant distance from healthcare relied on their willingness to travel.

P6: “You live in a place like that, you know you have to travel.”

There was also dependence on the specialist hospice nurse taking up part of this burden.

P2: “. . .the distances the hospice nurses drive. . .just immense. . .”

Care at end of life was identified as very challenging.

P8: “. . .it is tough, tough going.”

The transition from relative to carer was also demanding with changes in roles and relationships.

P16(1): “So, in the end, I became the parent. . . .and I had to say to her ‘for fuck sake mum, just fucking do as you’re told.”

Adaptation to living in remote rural conditions was seen as important by participants. This was achieved in several ways.

P4: “You know, everything needs. . .a lot more organising. . .”

Additionally, there was acceptance of the situation and a culture of resilience in end-of-life care; fostered by the experience of a perceived robust, rural life.

P10: “So I was trained to give her the morphine. . .injection. . .

I: . . .How was that for you?

P10: I’ve been farming so it was no issue.”

Other ways in which people adapted to caring for the dying were by relying on family and the local rural community to help.

P1: “You’ve got to have family. . .prepared to help. . .and neighbours. . .”

Communities were perceived as close-knit and would rally together to fill in gaps in healthcare.

P1: “You know, they’re always fundraising for stuff. . .and town, when we need something, they start fundraising. . .”

The specialist hospice service was also flexible in its care. Telehealth was part of this.

P3: “So, the next thing we are sitting up. . .on the bed, having a Zoom call with the hospice doctor in Waikato.”

Twenty-four-hour access to specialist Hospice input was also greatly appreciated.

P4: “My understanding of hospice is that they were always there for us, 24-hours a day on the phone, which, was once again, invaluable. . .”

### Positive nursing characteristics

The specialist hospice team, particularly the nurses, were seen as facilitators of meaningful moments. Participants also described positive experiences in terms of a holistic, person-centred approach.

There was an appreciation that professional care received helped to promote quality of life.

P3: “We’d sit there and have a drink at night; she’d have a little whiskey or something. We were able to. . .take her in the golf cart. . .we whisked around and did all sorts of things and. . .I think that it enabled us to. . . have a pretty full life. . .”

Specialist hospice nurses provided an empathetic, listening ear to participants and patients. Participants felt this was above and beyond professional duties.

P18: “Nothing was too much trouble. . .you could talk to them about anything. Even if it was the. . .silliest little thing. . .”

The hospice team were skilled at gaining trust and building positive relationships with patients and participants.

P18: “Gaining mum’s trust. . .it was the way that the hospice staff. . .were able to turn mum’s mindset around. . .they were. . .there to help her and help us to help her. . .it was the subtleness, it was the professionalism of it all and the warmth and the understanding of circumstances. . .”

### Hospice nurse role

The role of the rural hospice specialist nurse as a key communicator in the healthcare system was also highlighted as vital to good palliative care. This encompassed them as a collaborator and coordinator.

There were conflicting experiences for participants in terms of who to contact when they needed support.

P17: “You get a bit confused. . .. it was hard, it was. . .just understanding what the role was of each person. . .”

Other participants experienced the specialist hospice nurse taking on the role of being the key coordinator of care making things more straightforward.

P3: “I think that key person is really important. . .”

Good communication between healthcare teams was deemed necessary.

P3: “Communication that they had between themselves. . .That made it all happen. . .”

The role of signposting care at the end of life was highlighted as important by participants. Some felt they were well prepared by the hospice team.

P1: “. . .[de-identified] gave me a booklet from hospice, OK, this is when [de-identified] was sick and it was really, really. . .helpful. . .especially knowing what to expect when [de-identified] was actually dying.”

Another participant felt that this part of care could have been improved upon.

P23: “Nobody spoke to you about death and dying and

I: What to expect, or?

P23: No, no we didn’t have that conversation.”

### Strategic ideas

The participants suggested ideas about how specialist hospice services could be improved for rural patients and caregivers.

Resources were seen as lacking at times. Access to equipment such as beds, commodes, or walking aids was highlighted as an issue.

P6: “I ended up doing a lot of kilometres picking up bits and pieces when there could’ve been a slightly easier system. . . put in place.”

There was a lack of support from specialist hospice services in rural areas at weekends.

P18: “What I’m saying is, that if you had that hospice nurse there over the weekend that calls and checks on the patient as well. . .it’d probably give your family. . .peace of mind as well.”

Similarly, more frequent visits by the hospice team would be appreciated.

P11: “And perhaps. . . coming more often. . .”

Further support services such as access to respite care were highlighted.

P8: “I think respite care would probably. . .be good. . .”

More training for caregivers was also felt to be something that would be useful.

P12: “Yeah. . .some training, I think that would go a long way. . .teaching whānau how to look after their whānau.” (family)

Financial incentives were suggested as a way of promoting healthcare professionals to live in rural communities, which would facilitate a better understanding of the culture.

P2: “One of the problems is that for doctors. . .and quite a few of the nurses, they don’t come from small towns. They come from the big cities. . .So they have no idea what small town living is like. . .one way that you could attract them into it is to. . .allow a 10% reduction off their student loan for each year, for three years’ worth. . .of service in a rural community.”

Finally, emotional care and bereavement support were emphasised as areas where more resources are required.

P11: “. . .I think probably just the emotional support, myself. . .is the big thing for me.”

## Discussion

We researched carer perceptions of specialist hospice, palliative care in a range of rural communities. This study emphasises that culture within these communities is important in that rural residents, and the model of care must adapt to each other.

Robinson et al. interpreted carer resourcefulness and a perceived “duty” to become a caregiver in the rural setting. Increased sacrifice as care needs became greater, including driving very long distances led to neglect of caregiver self-care.^21^ The existing community support networks and rural community resilience have been felt to be facilitators of rural palliative care, but these coping strategies may lead to “making do”; a reluctance to seek professional help and heavier caregiver burden.^1^

A qualitative exploration of a “good death” as perceived by rural patients and caregivers in Australia concluded that they were prepared or forced to compromise to ensure safety in dying. This meant moving from isolated situations to places such as rural hospitals or age residential care facilities for end-of-life care.^22^

Our study confirms a willingness to compromise on the part of the rural carer; an acceptance that being a rural resident means less access to care and more burden on the carer.

However, our study gained insight into how carers perceive changes that could be made to enhance support in the rural setting. There was an acknowledgement that they had to be more flexible and organised but also thought that they should be met halfway by hospice services. This included a perceived need for more, better structured, or redistributed resources. During this research, one rural hospice nurse set up a local equipment repository in one of the rural communities with great success.

One rural caregiver voiced ideas that hospice service providers should reside within the rural community and provide care from within. This has also been identified by healthcare professionals as important to provide good quality rural palliative care.^8^

Strategies that caregivers felt would support them better were; more frequent visits from specialist hospice team members, access to respite care, more prominent emotional support and increased education about end-of-life care.

Our study also describes the role of the rural hospice nurse. This was defined by the interviewed carers, both in terms of existing positive personal characteristics, but also ways in which the position could be developed. Studies have previously identified the hospice nurse specialist as “confidante” and advocate, in semi-structured interviews of carers.^9,23^

Literature highlights the communication between healthcare professionals of involved services as fundamental to good rural palliative care, and in nursing.^8,24^ Hawkins-Taylor et al. looked at perceptions of palliative care in rural South Dakota. This involved focus groups with a mix of healthcare professionals and patients/carers and identified communication between them as key to success.^10^

Barlund et al. also undertook individual interviews with bereaved carers and reiterated this. They highlighted that having a well-organised healthcare system was necessary to make caregivers feel secure.^25^

Our study corroborates this, and in addition, highlights the carer perception that the rural hospice nurse could fulfil the role of coordinator of care between services, patient, and carer.

Educating carers on what to expect and how to care for a relative was seen as an important part of the role of the specialist hospice nurse – one which could be more consistent in the information and education provided. Education to rural primary healthcare providers must also be attended to, as it is known that generalist rural community healthcare providers lack palliative care expertise.^26^

Research has highlighted gaps in the knowledge of non-specialist rural community-based nurses in Australia concerning the provision of psychosocial care for palliative and end-of-life clients and their caregivers.^27^ Positive aspects of specialist hospice nursing care in our study underlined emotional support as part of the holistic care being provided. However, some rural carers also felt that this was lacking, demonstrating the absence of consistency of support across rural communities.

## Strengths and limitations

The key strength of this study is that it focused directly on key stakeholders’ (bereaved caregivers’) perceptions of rural specialist hospice care – a group very sparsely represented in the existing literature. Semi-structured, individual interviews allowed these “experts” to speak freely and give their views. However, interviewers commented that the telephone interviews lacked the same rapport which may have affected the information gathered.

The number of interviews and volume of data facilitated confidence that data saturation was achieved. The rural communities sampled were from a large region and diverse in culture and setting. Indigenous Māori were well represented, although this fell short of the MRRC recommendation to aim for 50% Māori participants. Reasons for this are likely to be multi-factorial.

It can be argued that “expert” sampling can create bias. For example, nurses may have selected participants they had positive relationships with. Opportunities to interview those with more negative experiences may have been missed. The study is based on one hospice, so carer perceptions gleaned are limited to the model of care provided by that particular service.

Patient perceptions in addition to carer views may have added another dimension to the data collected in this research.

Rural communities are diverse, often with their own micro-cultures. The views obtained were from a variety of such areas, but the data from each were analysed together. It must be acknowledged that there may not be a general approach to specialist hospice care that fits all rural settings.

## Conclusion

Table 4 shows the key themes, conclusions, and recommendations drawn from the analysis.

**Table 4.** Themes, conclusions, and recommendations.

Theme	Conclusions	Recommendations
Rural culture	“Rural resilience” as both a facilitator and barrier to successful palliative care.	Explore different models of care in rural communities to compare stakeholder perceptions of these.
Positive nursing characteristics	Specialist hospice nurses seen as facilitators of meaningful moments. Participants also described positive experiences with a holistic, person-centred approach.	Celebrate and feedback on carer perceptions of excellent holistic care. Use these descriptions as desirable traits for future recruitment of team members.
Hospice nurse role	Specialist hospice nurse seen as a coordinator, collaborator, and communicator, providing both emotional and holistic care.	Explore formalising key healthcare navigator as part of the specialist hospice nursing role.
Strategic ideas	Access to respite care, consistent carer education, timely access to equipment, having specialists embedded in communities with 7-day working, and increased emotional/bereavement support.	Discuss and implement access to respite care. Consider local equipment stores. Explore care models for both medical and nursing specialists in terms of support to primary care providers. Look at pathways to provide better psychological support. Plan for and facilitate more consistent carer training.

Rural caregivers are willing to accept some disparity in care because of their location and existing “rural resilience.” In contrast, they also felt that they deserved more flexibility and resources in palliative and end-of-life care in their communities. This would mean that they, their family, and neighbours are less relied upon.

Importantly, our study highlights the need to explore the balance between tailoring care to different rural communities against providing consistent, equitable care. This may be explored by studying different models of care in rural communities to compare stakeholder perceptions of these.

The bereaved caregivers identified the specialist hospice nurse role as one of a coordinator, collaborator, and communicator as well as providing both emotional and holistic care. There was a desire for a key navigator of the healthcare system; the specialist hospice nursing role could fulfil this if resources allow. However, it must be recognised that spreading this resource too thinly may result in unsustainable workload and professional burnout. A question not answered by our study is: how big a part of the hospice nursing role should emotional support be? Specialist psychological services may be more appropriate.

Respite care, consistent carer education, timely access to equipment, and having specialists embedded in communities with 7-day working were strategic ideas.

## Supplemental Material

sj-docx-1-pcr-10.1177_26323524251332970 – Supplemental material for Caregiver experience of specialist hospice palliative care in rural communities: A qualitative study exploring rural culture, hospice nurse characteristics, roles, and carer strategic ideas Supplemental material, sj-docx-1-pcr-10.1177_26323524251332970 for Caregiver experience of specialist hospice palliative care in rural communities: A qualitative study exploring rural culture, hospice nurse characteristics, roles, and carer strategic ideas by Rowan Daniel Beaumont Bell, Caren Amanda Barnett, Matthew Joseph Wilson and Philippa Marie Twigg in Palliative Care and Social Practice

## Statements and declarations

**Ethical Considerations:** The study was approved by the Southern Health and Disability Ethics Committee on 27 August 2023. Ethics reference: 2023 EXP 18102. Health New Zealand, Te Whatu Ora Waikato, and Te Puna Oranga Māori Research Review Committee (MRRC) consultation endorsed the research on 23 May 2023.

**Consent for Publication:** Was included as part of the consent information provided to participants at the outset of the study.

**Data Availability:** Supplementary materials can be accessed with a request to the corresponding author.
